# Type 2 diabetes subtypes, aetiology and pathophysiology. Reply to Vaag A [letter]

**DOI:** 10.1007/s00125-026-06888-y

**Published:** 2026-09-30

**Authors:** Tim Mori, Pedro Cardoso, John M. Dennis, Oliver Kuß

**Affiliations:** 1 Department of Medical Statistics, University Medical Center Göttingen, Göttingen, Germany; 2 Institute for Biometrics and Epidemiology, German Diabetes Center, Leibniz Center for Diabetes Research at Heinrich Heine University Düsseldorf, Düsseldorf, Germany; 3 German Center for Diabetes Research (DZD), Partner Düsseldorf, München-Neuherberg, Germany; 4 Institute of Clinical & Biomedical Sciences, University of Exeter Medical School, Exeter, UK; 5 Centre for Health and Society, Faculty of Medicine, Heinrich Heine University Düsseldorf, Düsseldorf, Germany

**Keywords:** Classification uncertainty, Clusters, Limitations, Methodology, Precision medicine, Prediction models, Review, Subtypes, Type 2 diabetes mellitus

*To the Editor*: We thank Dr Vaag [1] for his insightful comments regarding our recent publication, ‘Type 2 diabetes subtypes for precision medicine: methodological challenges and alternative prediction-based approaches’ [2]. Our narrative review focused on methodological limitations of data-driven diabetes subtypes [3] and argued that individualised prediction models [4] might be more useful for precision treatment (i.e. choosing the right treatment) and precision prognostics (i.e. predicting clinical outcomes). Dr Vaag’s letter extends these arguments by also challenging the subtypes’ utility for pathophysiological insights and precision diagnostics (i.e. refining the characterisation of the diabetes diagnosis) [1].

As statisticians and epidemiologists, we appreciate Dr Vaag’s biologically and clinically grounded criticism of the diabetes subtypes [1]. We agree that genetic differences between subtypes may be driven by differences in clinical features used for classification (e.g. HOMA), rather than reflecting distinct aetiologies. From a methodological perspective, the challenge of closely interlinked HOMA-IR and HOMA-B indices was also investigated in the simulation study by Aoki et al [5]. They found that the correlated HOMA measures could lead to the identification of spurious clusters when applying data-driven clustering methods, further suggesting a limited potential for pathophysiological insights. In contrast to this, recent individualised prediction models [4] have shown promising signs of clinical utility without using HOMA indices, instead relying on more straightforward and readily available clinical features (e.g. diabetes duration, eGFR).

Regarding precision diagnostics, Dr Vaag argues that, instead of focusing on these diabetes subtypes, further innovative approaches are needed to delineate the complex aetiology and pathophysiology of cardiometabolic diseases [1]. Indeed, an important question is how, and whether, the field should proceed with diabetes subtyping. Growing evidence on clinical [6, 7] and methodological [2] limitations raises doubts about whether data-driven subtyping based on previously published algorithms [3] is the most promising path forward. Indeed, as early as 2006, Gale [8] cautioned against an excessive focus on disease classification, arguing that ‘Our routine attempt to assign patients to one or other type of diabetes is (…) neither entirely rational, nor measurable, nor useful’. In line with this perspective, current evidence suggests that greater gains may be achieved by further developing and validating individualised prediction models [4], as also emphasised by the second Precision Medicine in Diabetes Initiative (PMDI) consensus report [9]. Nonetheless, if researchers choose to continue working with diabetes subtypes, it is important to remain mindful of their limitations, which we aimed to discuss in our narrative review [2].
